# Treatment of Sporadic Acute Puerperal Mastitis

**DOI:** 10.1155/S106474499600021X

**Published:** 1996

**Authors:** W. David Hager, John R. Barton

**Affiliations:** 1 Department of Obstetrics and Gynecology University of Kentucky Medical Center 800 Rose Street Lexington KY 40536-0084 USA; 2 Department of Obstetrics and Gynecology University of Kentucky Medical Center Central Baptist Hospital Lexington KY USA

## Abstract

*Objective:* The purposes of this study were to compare the efficacy of amoxicillin and cephradine for the treatment of sporadic acute puerperal mastitis (SAPM) and to evaluate the microbiology and clinical parameters of this infection.

*Methods:* We conducted a prospective, randomized, single-blinded study comparing amoxicillin, 500 mg orally q 8 h for 7 days, and cephradine, 500 mg orally q 6 h for 7 days. The diagnostic criteria for SAPM included a temperature of ≥37.56℃ (≥99.6℉) and erythema and tenderness of the breast(s).

*Results:* Twenty-seven consecutive outpatients with SAPM were evaluated for admission to the study, and 25 of these were enrolled. The mean temperature at enrollment was 38.17℃ (100.7℉), with a mean WBC count of 11,440/μl. The most frequent bacterial isolates from expressed milk were *Staphylococcus aureus* (7), staphylococcal species (coagulase negative) (8), and α-hemolytic streptococci (4). There were no significant differences between the 2 antibiotic regimens in cure rate, mean days to resolution of symptoms, or recurrence within 30 days. Both of the treatment failures and 1 of the 3 recurrences within 30 days were amoxicillin-treated patients whose cultures grew *S. aureus*.

*Conclusions:* Oral amoxicillin and cephradine appear equally effective in the treatment of SAPM. Staphylococci were the most frequent isolates from the milk of women with mastitis.


Infectious Diseases in Obstetrics and Gynecology 4:97–101 (1996)
© 1996 Wiley-Liss, Inc.

### Treatment of Sporadic Acute Puerperal Mastitis

**W. David Hager and John R. Barton**

*Departments of Obstetrics and Gynecology, University of Kentucky Medical Center, University of Kentucky School of Medicine (W.D.H.), and Central Baptist Hospital (J.R.B.), Lexington, KY*

### Abstract

*Objective:* The purposes of this study were to compare the efficacy of amoxicillin and cephradine for the treatment of sporadic acute puerperal mastitis (SAPM) and to evaluate the microbiology and clinical parameters of this infection.

*Methods:* We conducted a prospective, randomized, single-blinded study comparing amoxicillin, 500 mg orally q 8 h for 7 days, and cephradine, 500 mg orally q 6 h for 7 days. The diagnostic criteria for SAPM included a temperature of ≥37.56°C (≥99.6°F) and erythema and tenderness of the breast(s).

*Results:* Twenty-seven consecutive outpatients with SAPM were evaluated for admission to the study, and 25 of these were enrolled. The mean temperature at enrollment was 38.17°C (100.7°F), with a mean WBC count of 11,440/μl. The most frequent bacterial isolates from expressed milk were *Staphylococcus aureus* (7), staphylococcal species (coagulase negative) (8), and α-hemolytic streptococci (4). There were no significant differences between the 2 antibiotic regimens in cure rate, mean days to resolution of symptoms, or recurrence within 30 days. Both of the treatment failures and 1 of the 3 recurrences within 30 days were amoxicillin-treated patients whose cultures grew *S. aureus.*

*Conclusions:* Oral amoxicillin and cephradine appear equally effective in the treatment of SAPM. Staphylococci were the most frequent isolates from the milk of women with mastitis.

Key words

Breast infection, staphylococci, cephradine, amoxicillin, breast-feeding

Sporadic acute puerperal mastitis (SAPM) has been a recognized complication of breast-feeding since the process began. In 1953, Gibberd[1] categorized puerperal infections of the breast as epidemic and nonepidemic (sporadic). Epidemic mastitis is defined as acute adenitis and cellulitis primarily involving the lactiferous apparatus of the breast and often including nonadjacent lobes. It is usually seen in hospital outbreaks with transmission among mothers, babies, and hospital personnel. Most cases are caused by virulent strains of *Staphylococcus aureus.*[1,2] Nonepidemic mastitis is defined as acute puerperal cellulitis with extension to the periglandular connective tissue. This connective-tissue infection results in a V-shaped area of involvement among the lobes.

*S. aureus,* staphylococcal species (coagulase negative), group B streptococci, enterobacteriaceae, and anaerobic bacteria have been isolated from the milk of mothers with mastitis.[3,4] There are also data that potentially pathogenic bacteria may be isolated from the milk of nursing mothers who are not clinically infected.[5]

Actually, mastitis is a clinical and pathologic term that describes any inflammatory condition of the breast. The most frequent of these conditions is puerperal mastitis, an infection of the breast producing localized tenderness, erythema, and heat together with flu-like symptoms. Modern studies have reported the incidence of SAPM to be 2.5–2.9% of lactating mothers.[5,6] Although reviews and descriptive articles about puerperal mastitis are nu-

Address correspondence/reprint requests to Dr. W. David Hager, Department of Obstetrics and Gynecology, University of Kentucky Medical Center, 800 Rose Street, Lexington, KY 40536-0084.
Presented at the 22nd Annual Meeting of the Infectious Diseases Society for Obstetrics and Gynecology.

Received February 12, 1996
Accepted June 5, 1996

Brief Report merous,[4,7–9] few well-designed, prospective trials have focused on the epidemiologic aspects, etiology, and treatment of SAPM. The antibiotics studied have included β-lactamase-susceptible penicillins, penicillinase-resistant penicillins, cephalosporins, erythromycin, trimethoprim/sulfamethoxazole, and metronidazole.[7,10,11] The purpose of this study was to determine the clinical parameters, bacterial etiology, and response to antibiotic treatment of a cohort of mothers with SAPM. Amoxicillin and a cephalosporin were compared because these 2 antibiotics are most frequently prescribed to treat mastitis by members of the Infectious Diseases Society for Obstetrics and Gynecology.[12]

### MATERIALS AND METHODS

In this prospective, randomized, single-blinded study, a cohort of women with SAPM delivered by a single physician (W.D.H.) was randomized to treatment. The diagnostic criteria for SAPM were an oral temperature of ≥37.56°C (≥99.6°F) plus tenderness to palpation of the breast and segmental erythema.[7] All 3 criteria were required for the diagnosis of SAPM. The exclusion criteria included maternal age of <18 years, documented allergy to penicillins or cephalosporins, and antibiotic therapy within the previous 30 days.

The candidates for the study were lactating women who presented to the outpatient clinic with complaints of fever and breast discomfort. Upon examination, if the clinical criteria for diagnosis were met and no exclusion criteria were noted, the patient was offered participation in the study. If she agreed, an informed consent was signed. Historical information and study data were recorded on precoded data sheets.

After the areola was prepped with betadine, a specimen for cultures was obtained by expressing milk from the involved breast(s). After 3–4 drops were discharged, the milk was aspirated, placed onto transport media (modified Stuart's and modified Amies), and taken immediately to the laboratory for aerobic and anaerobic cultures, respectively. A Gram's stain was done on each isolate. The cultures were reported as mild, moderate, or heavy growth. A venous blood sample was also obtained for a CBC and WBC differential.

The study patients were randomized to 1 of 2 oral antibiotic regimens using presealed, opaque envelopes. The investigators were blinded to the antibiotic used. The treatment regimens were oral amoxicillin, 500 mg q 8 h for 7 days, or oral cephradine, 500 mg q 6 h for 7 days. Each patient was asked to continue breast-feeding and to apply warm, moist compresses to the involved breast(s) q 4–6 h. She was instructed to notify the physician if her temperature remained >37.56°C (>99.6°F) after 48 h or if she was unable to comply with the antibiotic regimen.

The study patients were seen for follow-up visits in 7 days. The symptoms and signs of disease were recorded, and an assessment of immediate cure or failure was made based on a resolution of the 3 principal diagnostic criteria: fever, erythema, and tenderness. A repeat CBC was drawn. If the patient had not responded to treatment, repeat cultures were obtained. The women who responded to treatment but subsequently developed findings of SAPM within 30 days were considered recurrences.

A statistical analysis was carried out using the Fisher's exact test (2-tailed). The differences were considered significant at $P < 0.05$.

### RESULTS

From July 1, 1991, until December 31, 1993, 566 women were delivered. Of those, 380 (67.1%) nursed their infants. Twenty-seven mothers (4.8%) who breast-fed presented with clinical findings of SAPM and 25 of them consented to participation in the study. One patient declined enrollment in the study and 1 took antibiotics within the previous 2 weeks.

Thirteen patients were randomized to treatment with amoxicillin and 12 to treatment with cephradine. There were no significant differences between the 2 groups in days to onset of infection, duration of symptoms before diagnosis, presence of cracked nipples at the time of diagnosis, bilateral involvement, mean temperature at diagnosis, or mean WBC count at diagnosis (Table 1). There were also no differences in mean age, parity, history of mastitis, or history of diabetes mellitus.

All study patients met the entry criteria of a temperature of ≥37.56°C (≥99.6°F) and erythema and tenderness of the breast(s). The mean time from delivery until the diagnosis of SAPM was 55.4 days. The mothers presented for care an average of 47.4 h after the symptoms began. Twenty (80%) had edema of the breast(s) and 16 (64%) had cracked nipples (10 in the amoxicillin group and 6

TABLE 1. Clinical parameters (means) of SAPM

| | Amoxicillin (N = 13) | Cephradine (N = 12) | Total |
|---|---|---|---|
| Days to onset | 56.5 | 54.3 | 55.4 (4–259) |
| Duration (h) of symptoms before diagnosis (range) | 39.6 (12–72) | 52.4 (12–168) | 47.4 (12–168) |
| Nipple cracked | 10 (77%) | 6 (50%) | 16 (64%) |
| Bilateral involvement | 1 | 1 | 2 (8%) |
| Mean temperature (range) | 38.17°C (37.56–39.72) 100.7°F (99.6–103.5) | 38.11°C (37.56–39.44) 100.6°F (99.6–103) | 38.17°C (37.56–39.72) 100.7°F (99.6–103.5) |
| Mean WBC count (× $10^9$/l) (range) | 11.9 (5.2–16.8) | 10.9 (6.8–16.1) | 11.4 (7.8–16.8) |

TABLE 2. Bacteriology (principal isolates) of SAPM

| | |
|---|---|
| *S. aureus* (PCN-R, Ceph-S)[a] | 7 |
| Amoxicillin-treated | 3 |
| Cephradine-treated | 4 |
| Staphylococcal species (PCN-R, Ceph-S) | 8 |
| Amoxicillin-treated | 4 |
| Cephradine-treated | 4 |
| α Streptococcus | 4 |
| Group B streptococcus | 2 |
| *Propionibacterium acne* | 1 |

[a]PCN-R = penicillin-resistant; Ceph-S = cephradine-sensitive.

in the cephradine group). The mean temperature at admission of all patients was 38.17°C (100.7°F) and the mean WBC count was 11,440/μl.

The bacteria isolated from the milk of patients are listed in Table 2. Staphylococci were the most frequent isolates. All of the staphylococcal isolates were resistant to penicillin and sensitive to cephalosporins. The coagulase-negative staphylococci were not speciated. Each of the positive cultures had moderate or heavy growth of the principal isolate.

There were 2 treatment failures in the amoxicillin group, 1 of whom developed a unilateral breast abscess. This patient had no clinical evidence of an abscess at the time of the initial diagnosis. One of these treatment failures had cracked nipples (the abscess patient) and the other did not. The cultures of both treatment failures grew *S. aureus* from their breast milk. The mother with the abscess was treated with incision and drainage of the abscess and a parenteral cephalosporin. A culture of the abscess taken at the time of the procedure grew *S. aureus*. The other treatment failure received oral cephradine and responded readily. Her cultures also grew *S. aureus* at the time of retreatment. There were no failures in the cephradine group (Table 3).

There was 1 recurrence within 30 days of treatment in the amoxicillin group. This patient's admission culture grew *S. aureus* and her culture at the time of the recurrence grew the same organism. There were 2 recurrences within 30 days in the cephradine group, with both culturing coagulase-negative staphylococcal species at admission to the study. The cultures at the time of recurrence on both of these patients were negative (Table 3).

One patient in each group had bilateral breast involvement, including the patient with the unilateral abscess. There were no adverse side effects to the antibiotics administered. The women were questioned about compliance with dosing at the return visit, and all indicated that they had taken their medication as prescribed.

### DISCUSSION

SAPM is an infection of the breast that complicates breast-feeding in 2.5–2.9% of women, according to modern studies.[5,6] Although 1 report indicated that mastitis occurred at least once in approximately one-third of mothers during a single lactation course, this study was a retrospective analysis by questionnaire depending on recall for data.[8] In our prospective analysis, the symptoms of SAPM caused 4.8% of 380 nursing mothers to seek care.

Various clinical parameters have been evaluated in other studies.[5,13,14] It has been found that 67% of patients have the onset of symptoms in the first 6 months of nursing. The mean time to onset of symptoms was reported to be 38 days. Both breasts were involved 8–10% of the time. A recurrence of

TABLE 3. Results of SAPM

| | Amoxicillin (N = 13) | Cephradine (N = 12) |
|---|---|---|
| Treatment failures* | 2 (*S. aureus*) (15.4%) | 0 |
| Days to resolution of symptoms* (range) | 4.2 (1–5) | 3.8 (1–4) |
| Abscess* | 1 | 0 |
| Recurrences within 30 days* | 1 (*S. aureus*) | 2 (coagulase-negative staphylococcal species) |
| Bilateral involvement | 1 | 1 |

*$P$ = NS (Fisher's exact test).

mastitis occurred within 30 days of the cessation of treatment in 8–10% of patients. An abscess complicated the acute process in 1–11% of nursing mothers.

Thomsen et al.[15] demonstrated that the lactating breast is susceptible to infection by inducing mastitis in lactating mice with the inoculation of *S. epidermidis* or *S. saprophyticus* into their mammary glands. Other investigators have reported *S. aureus* to be the principal isolate in 48–50% of cases.[4,5] Other isolates have included coagulase-negative staphylococcal species, group B streptococci, Enterobacteriaceae, and anaerobic bacteria.

In our study of lactating mothers, *S. aureus* was recovered from the milk of 3 amoxicillin-treated women and 4 cephradine-treated women, for a total isolation rate of 28%. Staphylococcal species (coagulase negative) were isolated from the milk of 4 amoxicillin-treated and 4 cephradine-treated mothers, for a total isolation rate of 32%. These isolation rates are consistent with those referenced above. Since 60% of the mothers had staphylococci cultured from their milk, it seems prudent to treat women with SAPM with an antibiotic that is effective against these bacteria, if these bacteria truly infect the breast parenchyma.

All of the staphylococcal isolates were resistant to penicillin and sensitive to cephalosporins. Both treatment failures occurred in the amoxicillin group, and both patients' cultures grew *S. aureus* upon admission to the study and at the time of their assessments as treatment failures. One of these women developed a breast abscess; the other responded when her antibiotic was changed to cephradine. The culture of the amoxicillin-treated patient with a recurrence also grew staphylococcal species (coagulase negative) and she responded to treatment with cephradine.

Among the amoxicillin-treated group, 1 whose culture grew *S. aureus* and 4 whose cultures grew staphylococcal species responded to treatment in spite of the fact that the isolates were resistant in vitro. This finding may emphasize the importance of emptying the breast and using warm, moist compresses in the treatment of SAPM.

We recognize that the number of patients in this study is small. In a post hoc analysis, if we assume a 15% failure rate for amoxicillin and a 1% failure rate for cephradine, with an $\alpha$ of 0.05 and desired power of 0.8, 72 patients would be required for each arm of the study using a continuity correction for small numbers. Based on our recruitment of nursing mothers, a delivery population of 3,264 women would be required. We will continue to recruit more patients to achieve these numbers and will report those data.

There was no statistically significant difference in response to antibiotic treatment ($P > 0.05$) between the 2 regimens. The facts that both failures were initially treated with amoxicillin (a β-lactamase-susceptible penicillin), the abscess was found in an amoxicillin-treated patient, and all of the staphylococcal isolates were penicillin-resistant cause us to express our bias toward the recommendation of a β-lactamase-resistant antibiotic such as a cephalosporin along with the application of moist heat and continued breast-feeding.

### REFERENCES

1. Gibberd GF: Sporadic and epidemic puerperal breast infections. Am J Obstet Gynecol 65:1038–1041, 1953.
2. Marsh F: Staphylococcal infection in maternity hospitals. Lancet 2:1179–1180, 1948.
3. Carrol L, Davies DP, Osman M, McNeigh AS: Bacteriologic criteria for feeding raw breast milk to babies on neonatal units. Lancet 2:732–733, 1979.
4. Niebyl JR, Spence MR, Parmley TH: Sporadic (nonepidemic) puerperal mastitis. J Reprod Med 20:97–100, 1978.

5. Marshall BR, Heppler JK, Zirbel CC: Sporadic puerperal mastitis, an infection that need not interrupt lactation. JAMA 233:1377–1379, 1975.
6. Kaufmann J, Foxman B: Mastitis among lactating women: Occurrence and risk factors. Soc Sci Med 33: 701–705, 1991.
7. McGregor JA, Neifert MR: Maternal problems in lactation. In Neville MC, Neifert MR (eds): Lactation Physiology, Nutrition and Breastfeeding. New York: Plenum Press, 334–337, 1983.
8. Riordan JM, Nichols FH: A descriptive study of lactation mastitis in long-term breastfeeding women. J Hum Lact 6:53–58, 1990.
9. Lawrence R: Management of mother-infant couple. In Lawrence R: Breastfeeding: A Guide for the Medical Profession. 4th ed. St. Louis: C.V. Mosby, 257–267, 1994.
10. Thomsen AC, Espersen T, Maigaard S: Course and treatment of milk stasis, noninfectious inflammation of the breast, and infectious mastitis in nursing women. Am J Obstet Gynecol 149:492–495, 1984.
11. Cantlie HB: Treatment of acute puerperal mastitis and breast abscess. Can Fam Phys 34:2221–2226, 1988.
12. Hager WD: OB-GYN infections: Bacterial causes and antibiotic choices. Contemp OB-GYN 29:121, 1987.
13. Devereux WP: Acute puerperal mastitis: Evaluation of its management. Am J Obstet Gynecol 108:78–81, 1970.
14. Thomsen AC, Hansen KB, Moller BR: Leukocyte counts and microbiologic cultivation in the diagnosis of puerperal mastitis. Am J Obstet Gynecol 146:938–941, 1983.
15. Thomsen AC, Mogensen SC, Love-Jepsen F: Experimental mastitis in mice induced by coagulase-negative staphylococci isolated from cases of mastitis in nursing women. Acta Obstet Gynaecol Scand 64:163–166, 1985.

## References

[PDF_00370] Cantlie H. B. (1988). Treatment of acute puerperal mastitis and breast abscess.. Can Fam Physician.

[PDF_00342] Carroll L., Osman M., Davies D. P., McNeish A. S. (1979). Bacteriological criteria for feeding raw breast-milk to babies on neonatal units.. Lancet.

[PDF_00374] Devereux W. P. (1970). Acute puerperal mastitis. Evaluation of its management.. Am J Obstet Gynecol.

[PDF_00338] GIBBERD G. F. (1953). Sporadic and epidemic puerperal breast infections; a contrast in morbid anatomy and clinical signs.. Am J Obstet Gynecol.

[PDF_00353] Kaufmann R., Foxman B. (1991). Mastitis among lactating women: occurrence and risk factors.. Soc Sci Med.

[PDF_00348] Marshall B. R., Hepper J. K., Zirbel C. C. (1975). Sporadic puerperal mastitis. An infection that need not interrupt lactation.. JAMA.

[PDF_00345] Niebyl J. R., Spence M. R., Parmley T. H. (1978). Sporadic (nonepidemic) puerperal mastitis.. J Reprod Med.

[PDF_00360] Riordan J. M., Nichols F. H. (1990). A descriptive study of lactation mastitis in long-term breastfeeding women.. J Hum Lact.

[PDF_00366] Thomsen A. C., Espersen T., Maigaard S. (1984). Course and treatment of milk stasis, noninfectious inflammation of the breast, and infectious mastitis in nursing women.. Am J Obstet Gynecol.

[PDF_00376] Thomsen A. C., Hansen K. B., Møller B. R. (1983). Leukocyte counts and microbiologic cultivation in the diagnosis of puerperal mastitis.. Am J Obstet Gynecol.

[PDF_00380] Thomsen A. C., Mogensen S. C., Løve Jepsen F. (1985). Experimental mastitis in mice induced by coagulase-negative staphylococci isolated from cases of mastitis in nursing women.. Acta Obstet Gynecol Scand.

